# Imaging cholesterol depletion at the plasma membrane by methyl-β-cyclodextrin

**DOI:** 10.1016/j.jlr.2021.100077

**Published:** 2021-04-21

**Authors:** Mitsuhiro Abe, Toshihide Kobayashi

**Affiliations:** 1 Lipid Biology Laboratory, RIKEN, Wako, Saitama, Japan; 2 Cellular Informatics Laboratory, RIKEN, Wako, Saitama, Japan; 3 Laboratoire de Bioimagerie et Pathologies, UMR 7021 CNRS, Université de Strasbourg, Faculté de Pharmacie, Illkirch, France

The most common method to modify cellular cholesterol (Chol) content in vitro is to incubate cells with cyclodextrin (CD). However, it is not well understood how Chol is removed by CD. Recent development of protein probes that bind Chol allowed to visualize and to follow cellular Chol semiquantitatively. In this image, nontoxic Chol-binding D4 fragment of bacterial toxin, perfringolysin O, was conjugated with mCherry (mCherry-D4) (1) and the protein was transiently expressed in HeLa cells for 48 h. The cells were synchronized with 40 ng/ml nocodazole for 3 h, and mitotic cells were harvested by shake-off. The harvested cells were plated in a poly-D-lysine–coated dish (BD) and further incubated for 30 min. Then, nocodazole was washed out and 10 μg/ml of recombinant enhanced green fluorescent protein-D4 (EGFP-D4) was added to the medium. After 10 min, we started to acquire fluorescence images every 5 min. After 80 min, 1 mM MβCD was added. Selected images are shown in Fig. 1. Accumulated images are shown in supplemental Videos S1 and S2. The quantitated data are shown as graphs in supplemental Fig. S1. Supplemental Figure S2 shows that equivalent amount of EGFP-D4 and mCherry-D4 displayed similar Chol concentrations dependent binding to phosphatidylcholine/Chol membranes. Under this condition, mCherry-D4 and EGFP-D4 bind accessible Chol (2) in the inner and outer leaflets of the plasma membrane, respectively (1). Owing to high threshold of Chol detection by D4, intracellular organelles were not labeled with mCherry-D4 and depletion of Chol was accompanied by the intracellular accumulation of the protein. MβCD induced the rapid decrease (5–15 min) of inner leaflet Chol below the D4-binding threshold followed by the slow depletion phase in both outer and inner leaflets. The interaction between D4 or MβCD and cultured cells is not fully elucidated. Although MβCD is endocytosed, endocytosis is inhibited in mitotic cells (3). Binding of acrylodan-labeled D4 to phosphatidylcholine/Chol membrane is inhibited by cytosol (4). In addition to Chol, MβCD is reported to interact with other lipids and protein components of the membrane. However, extraction of cytosolic protein by MβCD has not been reported. Our result suggests that the removal of outer leaflet Chol by MβCD is accompanied by the rapid flip of Chol from the inner leaflet to the outer leaflet. However, we cannot exclude the possibility that MβCD preferentially removed Chol from the inner leaflet.

**Figure 1:**
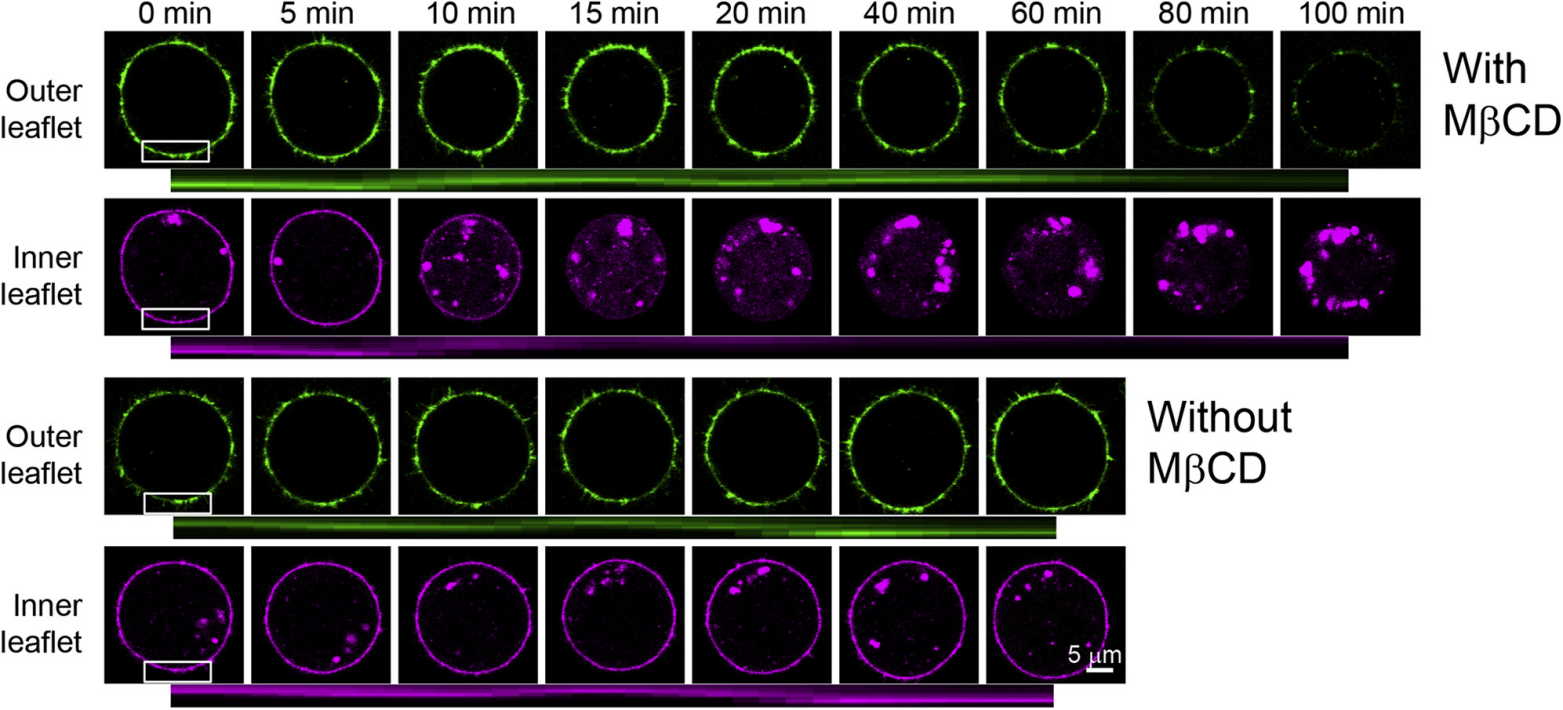
Fluorescence images were acquired in the absence of MβCD every 5 min (the starting time was taken as time 0 for −MβCD). After 80 min, 1 mM MβCD was added. This time point was taken as time 0 for +MβCD. Average fluorescence intensities of EGFP-D4 (*green*) and mCherry-D4 (*magenta*) were quantified in the area surrounded by the white rectangle and schematically shown in the kymographs below fluorescence images.

**EQUIPMENT:** FV 1000 confocal microscope with a 60x 1.1 NA Plan Apo objective lens (Olympus) equipped with an environmental chamber maintained with humidity, at 37°C and 5% CO_2_ and ImageQuant LAS 500 (Cytiva) were used.

**REAGENTS:** DMEM supplemented with 10% fetal bovine serum, Lipofectamine 3000 (Thermo Fisher Scientific), pET28/His6-EGFP-D4 (Catalog # RDB13961, Gene Engineering Division, RIKEN BRC), pET28/His6-mCherry-D4 (Catalog # RDB14300, RIKEN BRC), pDEST/mCherry-D4 (Catalog # RDB18944, RIKEN BRC), nocodazole (Sigma-Aldrich), MβCD (Cyclolab), and SYPRO Ruby (Thermo Fisher Scientific) were used.

## Supplemental data

This article contains supplemental data.

## Conflict of interest

The authors declare that they have no conflicts of interest with the contents of this article.
