# Supplementary figures and images for: Imaging cholesterol depletion at the plasma membrane by methyl-β-cyclodextrin
Source: J Lipid Res. 2021 Apr 21;62:100077. doi: 10.1016/j.jlr.2021.100077 (PMC8281586; doi:10.1016/j.jlr.2021.100077)

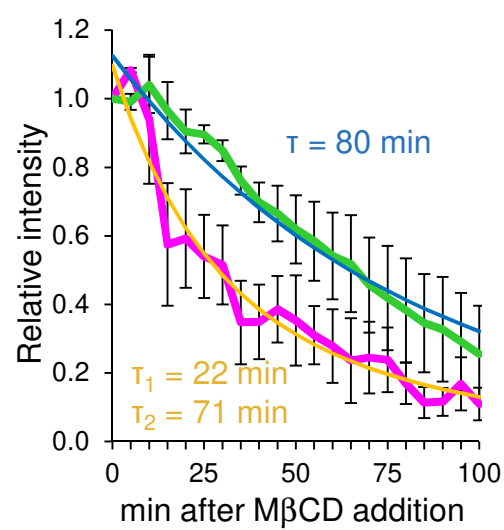

Supplement: Supplemental Figure S1 [file mmc3.pdf]

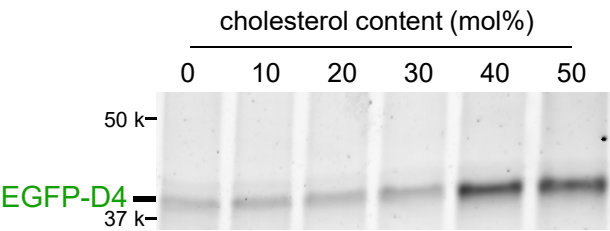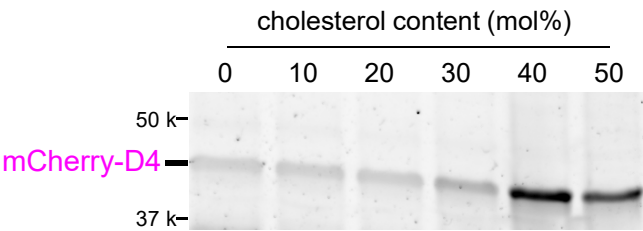

Supplement: Supplemental Figure S2 [file mmc4.pdf]
