# Supplementary material for: Imaging cholesterol depletion at the plasma membrane by methyl-β-cyclodextrin
Source: J Lipid Res. 2021 Apr 21;62:100077. doi: 10.1016/j.jlr.2021.100077 (PMC8281586; doi:10.1016/j.jlr.2021.100077)
Supplement: Supplemental Figure Legends [file mmc5.pdf]

SUPPLEMENTAL FIGURE LEGENDS

LEGEND FOR SUPPLEMENTAL FIGURE 1

Fluorescence intensities of EGFP-D4 (green) and mCherry-D4 (magenta) in the Figure were quantified in the area surrounded by white rectangle with ImageJ software (NIH) and fitted by a single exponential decay function (blue) or the sum of two single exponential functions (yellow) with MATLAB software (The MathWorks, Inc.).

LEGEND FOR SUPPLEMENTAL FIGURE 2

40 μg/ml of EGFP-D4 and mCherry-D4 were incubated with 1 mM multilamellar vesicles composed of 1,2-dioleoyl-sn-glycero-3-phosphocholine (DOPC) containing the indicated amount of Chol in PBS (pH7.5) for 30 min at room temperature. Then, the mixtures were centrifuged at 21,600 g for 10 min at 25°C. The pellets were subjected to SDS-PAGE followed by SYPRO Ruby staining.

LEGEND FOR VIDEO 1

Fluorescence images of EGFP-D4 (green) and mCherry-D4 (magenta) in the absence of MβCD. Images were captured at 5-min intervals and are replayed at 4 frames per second. Scale bar represents 5 μm.

LEGEND FOR VIDEO 2

Fluorescence images of EGFP-D4 (green) and mCherry-D4 (magenta) after addition of 1 mM MβCD. Images were captured at 5-min intervals and are replayed at 4 frames per second. Scale bar represents 5 μm.
